# Patients with postprandial distress syndrome experience problems with their interoceptive perceptual function to the gastric region, but their heartbeat perception is normal: a case control study

**DOI:** 10.1186/s13030-023-00290-5

**Published:** 2023-10-08

**Authors:** Kohei Yoshida, Tetsuya Abe, Kenji Kanbara, Kento Ueda, Yukie Saka-Kouchi, Hideaki Hasuo

**Affiliations:** 1 Department of Psychosomatic and General Internal Medicine, Kansai Medical University, 2-5-1-505 Shinmachi, Hirakata, 573-1010 Osaka Japan; 2 Psychosomatic Medicine, Department of Clinical Psychology, Kagawa University Faculty of Medicine, Kagawa, Japan

**Keywords:** Postprandial distress syndrome, Interoceptive accuracy, Heartbeat tracking task, Five-minute water load test, Visceral hypersensitivity

## Abstract

**Background:**

Visceral hypersensitivity in functional dyspepsia can be localized or widespread, and there is no simple method of assessment. Measuring interoceptive accuracy at different sites provides an assessment of perceptual hypersensitivity to specific ecological phenomena. The purpose of this study was to characterize visceral hypersensitivity by comparing gastric sensory and cardiac perceptual tests in patients with postprandial distress syndrome and in healthy volunteers.

**Methods:**

Sixteen patients with postprandial distress syndrome (age = 47.5 ± 17.4, all female) and 16 healthy volunteers (age = 43.3 ± 16.1, all female) participated in the study after a six-hour fast. Each participant answered questionnaires about physical and mental quality of life, depression and anxiety, tendency of alexithymia, and somatosensory amplification. After completing the questionnaire, the participants took the heartbeat tracking task and the five-minute water load test. We performed statistical analysis using the Mann–Whitney U test and Spearman’s rank correlation coefficient.

**Results:**

Subjects with postprandial distress syndrome had a lower drinking capacity than healthy volunteers (postprandial distress syndrome = 360.9 ± 170.0 mL, healthy volunteers = 644.1 ± 297 mL, P = 0.009), but there was no significant difference in the heartbeat perception score (postprandial distress syndrome = 0.599 ± 0.175, healthy volunteers = 0.623 ± 0.181, P = 0.647). There was a negative correlation (r = − 0.509, P < 0.05) between drinking capacity and the heartbeat perception score in healthy volunteers, but no correlation in postprandial distress syndrome (r = − 0.156, P = 0.564). Heartbeat perception score did not correlate with psychological measures.

**Conclusions:**

Compared with healthy volunteers, only the five-minute water load test values were reduced in patients with postprandial distress syndrome, and no difference was observed in the heartbeat tracking task. Combining the 5-minute water load test and the heart rate tracking task revealed a lost cardiac-gastric perceptual relationship in patients with postprandial distress syndrome that was not observed in healthy volunteers, suggesting that there is hypersensitivity in gastric interoceptive perceptual function. Performing sensory examinations at two different sites may be useful in clarifying whether visceral hypersensitivity is localized.

**Trial registration:**

UMIN000057586. Registered11 March 2023(retrospectively registered).

## Background

Interoception is internal body representation, which includes perceiving, interpreting, integrating, and regulating information about physiological states throughout the body [1, 2]. Interoceptive signals provide vital information to central control programs and contribute to the maintenance of homeostasis [3]. Interoception is composed of three dimensions: objective, subjective, and metacognitive aspects [4, 5]. Interoceptive accuracy (IAc) is the objective aspect of interoception and is the core component among its three dimensions [5].

The various interoceptive signals are delivered from the peripheral nerves to the central nervous system and integrated in the anterior insular cortex (AIC) [2, 6]. This process is separable by bodily axes, a set of single organs and their peripheral nerves and central controls [7], and the IAc dimension of each organ or peripheral site is fractionated across each bodily axis [7–9]. The gastric and cardiac axes are correlated, but the cardiac and respiratory axes are not related in the IAc dimension [8, 10, 11]. Measuring interoception across multiple bodily axes is an indicator of whether biological phenomena of one organ system are perceived more accurately than those of other organ systems and helps to capture the characteristics of an individual’s interoceptive process [7].

Visceral hypersensitivity (VH) is an important factor in symptom generation as part of functional gastrointestinal disorder (FGID) [12, 13]. In functional dyspepsia (FD), VH is present when there is hypersensitivity to gastric stretch stimuli, to capsaicin, and to acid [14–17]. VH is one of the explanatory variables of symptoms of postprandial distress syndrome (PDS) [18]. Several studies have reported conflicting results, with sensory hyperalgesia in FGID being limited to the symptom site [19–21] or occurring across other organs and body parts [17, 22]. It has been suggested that this difference may be caused by differences in whether central or peripheral factors are more pronounced within an individual [20]. When comparing the central disturbances of the subgroups in epigastric pain syndrome (EPS), increased central activity at rest is important in the pathogenesis [23]; PDS has been suggested to induce symptoms via central mechanisms in the stimulation of gastric advancement by drinking water, but central activity at rest is not important [23].

In FD, interoceptive disturbances in brain–gut signaling are thought to lead to hypersensitivity and visceral pain and discomfort [24]. However, VH and other symptoms in FD can be localized or widespread and vary from individual to individual. Although visceral perception has been the focus of much attention, studies of interoception in FGID have focused specifically on the site of symptoms, and there is a lack of research on how other organs are perceived. Previous studies have shown that VH is an explanatory variable for PDS symptoms, that PDS is induced by a central response to gastric stretch, and that there is a difference in central activity during rest between EPS and PDS. Based on these findings, we hypothesize that PDS is a problem of gastric axis interoceptive processes and involves only abnormal sensory processing in the stomach, not hypersensitivity across organs. The aim of this study was to characterize VH in PDS by examining the relationship between the cardiac axis IAc and gastric sensory perception, which is negatively correlated in healthy women with PDS.

## Methods

### Study design

This case control study was done from November 2018 through December 2019. Participants enrolled in the study without any compensation. The study was approved by the Ethics Review Board of the University Hospital (2,017,316).

### Participants

The patient group consisted of 16 women diagnosed with PDS and treated at the Department of Psychosomatic Medicine at a university hospital from November 2018 to December 2019. The diagnosis was made by a psychosomatic physician who treated the patient according to the Rome IV criteria. The control group consisted of 19 healthy women from the university who responded to recruitment posters. The exclusion criteria were age under 20 years, mental illness, and arrhythmia. The diagnosis of a psychiatric disorder was assessed by a psychosomatic physician who treated the patient according to DSM-V. In accordance with the study protocol approved by the Institutional Review Board, written informed consent was obtained from all participants. In the control group, one participant who could not do a task and two others for whom we were unable to measure the heart rate owing to technical difficulties were excluded, leaving the data of 16 healthy volunteers (HV) available for analysis.

### Procedure

Before the study, the participants stopped taking medications or alcohol for 24 h and had no caffeine for 12 h. After at least 6 h of fasting, they completed a self-administered questionnaire survey in a quiet room with the room temperature maintained at 25 °C in the morning. They then took the heartbeat tracking task (HTT) and the five-minute water load test (WL5) in that order.

### Measures

#### Heartbeat tracking task

The HTT is a highly reliable method of measuring IAc [5]. A wearable electrocardiograph device (myBeat heart rate sensor, WHS-2 Union Tool Co., Ltd., Tokyo, Japan) was used, and the Ag/AgCl adhesive disposable gel electrodes were affixed to the participants’ chests. Following Schandry’s mental tracking method, the participants were asked to close their eyes and count their heartbeats in random order for four intervals (each lasting 25, 35, 45, and 55 s) divided by 30-second breaks [10, 25]. Participants were instructed to place their hands on their thighs when counting heartbeats and were asked not to perform any actions that would facilitate heartbeat detection. Electrocardiograms were recorded during all the counting periods. The heartbeat perception score was calculated across all four intervals using the following conversion formula:

HTT score = 1/4Σ [1– (|recorded heartbeats – counted heartbeats|)/recorded heartbeats].

This score varies between 0 and 1, with 1 indicating a perfect match between the counted and recorded heartbeats.

#### Five-minute water load test

Drinking capacity in the WL5 test is used as a scale of gastric sensation [15]. This test has high reproducibility as an index of VH in patients with FD, and it also correlates positively with barostat test results [15]. While barostat testing is expensive and invasive [26], the WL5 is noninvasive because it uses natural expansion stimuli [26, 27], making it easy for clinical use. The experimental method followed that of Herbert et al. [10]. The participants were asked to consume water at a temperature of 25 °C ad libitum from a paper cup at a constant rate for at least 5 min until they reached the point of subjective fullness. The participants were instructed to stop drinking at the first signs of fullness. Behind a screen, the investigator kept refilling the cup out of sight of the participant. Therefore, the cup was “bottomless,” and each participant was blinded as to the amount of water they had consumed. The amount consumed was recorded by the end of the test.

#### Health-related quality of life (HRQoL)

HRQoL was measured with the Japanese version of the SF-8™ [28]. This item is scored in eight dimensions, comprising general health, physical functioning, role functioning—physical, body pain, vitality, social functioning, mental health, and role functioning—emotional. The physical component summary (PCS) and mental component summary (MCS) were calculated from these scores. Scores range from 62.6 to 19.6 for PCS and 62.7–14.8 for MCS. Higher PCS scores are assessed as higher physical health and higher MCS scores as higher mental health. The mean scores and standard deviations for the Japanese are as follows; PCS mean 49.84 ± 5.99 (Female mean 49.72 ± 6.09), MCS mean 50.09 ± 6.04 (Female mean 49.78 ± 6.05).

#### Anxiety and depression

Anxiety and depression were measured using the Hospital Anxiety and Depression Scale (HADS) [29]. This consists of seven items about anxiety (HADS-anxiety) and seven items about depression (HADS-depression). Each item is rated on a 0–3-point scale, and the total score range is 0–21-points. The cut-off point for clinical levels was set at a score > 11.

### Subjective somatic sensitivity

Psychological sensitivity to somatic symptoms was assessed using the Somatosensory Amplification Scale (SSAS) [30], in which 10 items were rated on a 1–5-point scale. A higher total score indicates a greater tendency to feel somatic sensations as uncomfortable and unpleasant.

### Alexithymia

Alexithymia was measured using the Toronto Alexithymia Scale of 20 items (TAS-20) [31]. These items are measured on a scale of 1–5 points, with the total score ranging from 20 to 100. A higher total score indicates a stronger tendency toward alexithymia.

### Data analysis

Data are presented as mean and standard deviation (SD). A Mann–Whitney U test was used to compare each score between the two groups. Spearman’s rank correlation coefficient was used to analyze the correlations between the scales in each group. P < 0.05 was regarded as significant in all analyses. The collected data were analyzed using PASW Statistics 18.0 for Windows TM (SPSS Inc., Chicago, IL).

## Results

### Characteristics of the participants

Table 1 shows the mean and SD of the participants’ age, height, weight, and body mass index. There were no statistically significant differences in any of these characteristics between the PDS and HV groups (P > 0.05).

**Table 1:** Participant demographics

	PDS			HV		P ^a^	
	N = 16			N = 16			
	Mean	(SD)		Mean	(SD)		
Age	47.5	(17.4)		43.3	(16.1)	0.584	
Height (cm)	155.7	(6.1)		158.3	(5.0)	0.416	
Weight (kg)	45.4	(9.5)		50.4	(5.8)	0.131	
Body mass index	18.8	(4.0)		20.2	(2.6)	0.546	

^a^ Mann–Whitney U test

PDS, postprandial distress syndrome; HV, healthy volunteers; SD, standard deviation

### Differences between PDS and HV groups in all measures

**Fig. 1:**
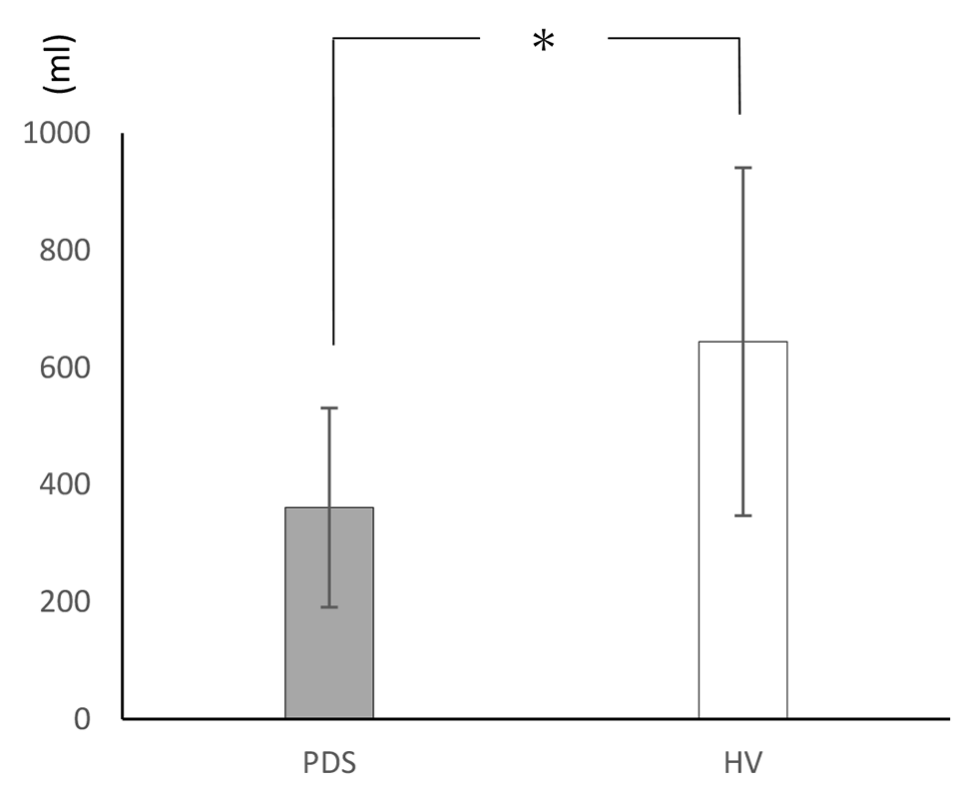
Drinking capacity (WL5)

**Fig. 2:**
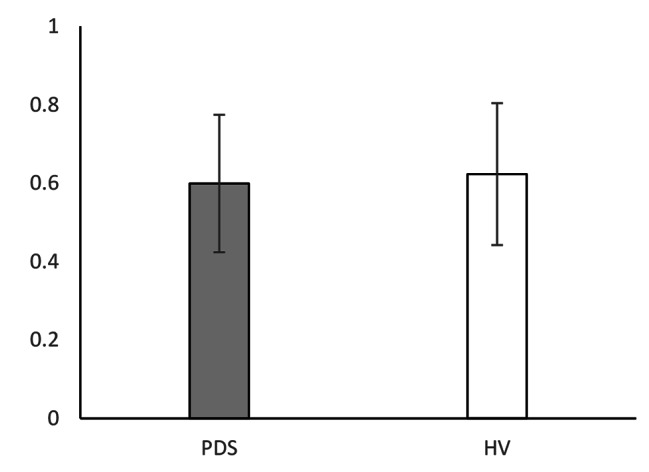
HTT score

**Fig. 3:**
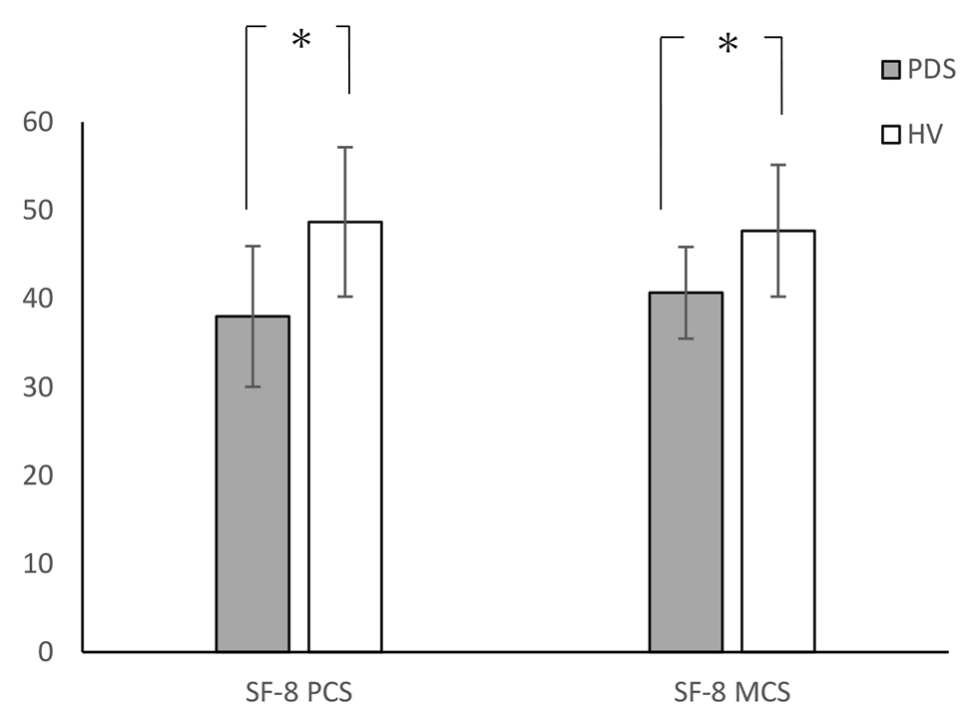
SF-8 score

**Fig. 4:**
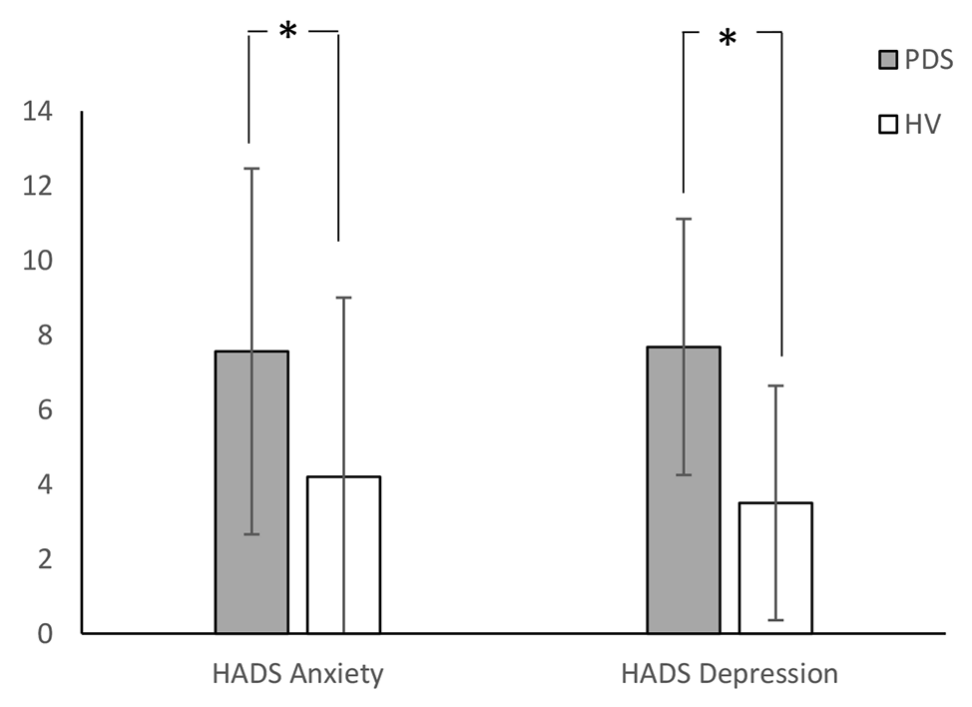
HADS score

**Fig. 5:**
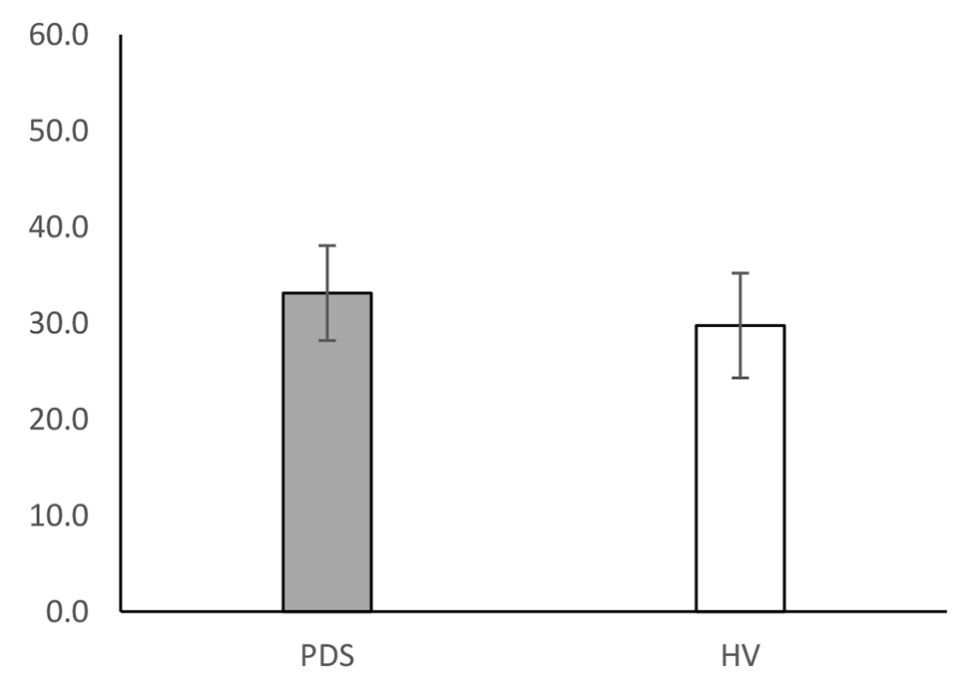
SSAS score

**Fig. 6:**
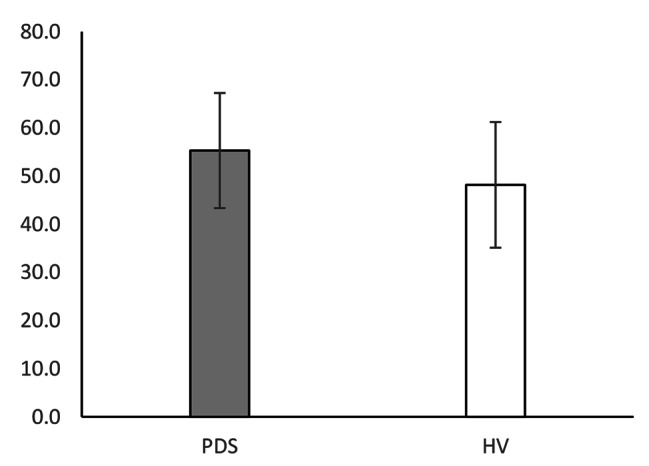
TAS-20 score

Table 2 shows the means, SDs, and differences of all measures. The WL5 values were significantly lower in the PDS group (PDS: mean 360.9 ± 170 mL; HV: mean 644.1 ± 297 mL; P = 0.009; Fig. 1). However, there was no difference in HTT scores between the PDS and HV groups (PDS: mean 0.599 ± 0.175; HV: 0.623 ± 0.181; P = 0.647; Fig. 2). With respect to HRQoL, both PCS (PDS: 38.0 ± 8.0; HV: 48.7 ± 5.2; P < 0.001) and MCS (PDS: 40.7 ± 8.5; HV: 47.7 ± 7.5; P = 0.017) were significantly lower in the PDS group compared with the HV group (Fig. 3). While the HADS-depression score was significantly higher in the PDS group than in the HV group (PDS: 7.7 ± 4.8; HV: 3.5 ± 3.1; P = 0.007; Fig. 4), HADS-depression and HADS-anxiety scores were within normal limits in both groups (cut off point: <11). SSAS and TAS-20 showed no significant differences between the groups (Figs. 5 and 6).

**Table 2:** WL5, HTT scores, and other measures: differences between PDS and HV

Variable	PDS			HV		P ^a^	
	(N = 16)			(N = 16)			
	Mean	(SD)		Mean	(SD)		
WL5 (ml)	360.9	(170.0)		644.1	(297.0)	0.009*	
HTT	0.599	(0.175)		0.623	(0.181)	0.647	
PCS	38.0	(8.0)		48.7	(5.2)	0.000*	
MCS	40.7	(8.5)		47.7	(7.5)	0.017*	
HADS-anxiety	7.6	(4.9)		4.2	(3.4)	0.067	
HADS-depression	7.7	(4.8)		3.5	(3.1)	0.007*	
SSAS	33.1	(4.9)		29.8	(5.4)	0.094	
TAS-20	55.3	(12.0)		48.2	(13.0)	0.094	

^a^ Mann–Whitney *U* test; * P < 0.05

PDS, postprandial distress syndrome; HV, healthy volunteers; SD, standard deviation; WL5, five-minute water load test; HTT, heartbeat tracking task; PCS, physical component summary; MCS, mental component summary; HADS, Hospital Anxiety and Depression Scale; SSAS, Somatosensory Amplification Scale; TAS-20, Toronto Alexithymia Scale of 20 items

### Correlation between WL5, HTT, and other measures in patients with PDS

Table 3 shows the correlation coefficients for each of the measured items within the PDS group. The WL5 and HTT scores were not correlated (r = − 0.156, P > 0.05). However, there was a strong positive significant correlation between WL5 and MCS scores (r = 0.762, P < 0.001), and a moderate negative significant correlation with the HADS-depression score (r = − 0.555, P < 0.05). The HTT was not correlated with any other measurement, including HADS-anxiety, in the PDS group.

**Table 3:** Spearman’s rank correlation between WL5, the HTT score, and other measures in patients with PDS

		WL5				HTT		
		r	P ^b^			r	P ^b^	
HTT		− 0.156	0.564					
PCS		0.426	0.099			− 0.176	0.513	
MCS		0.762	0.001*			0.000	1.000	
HADS-anxiety		− 0.378	0.149			0.094	0.728	
HADS-depression		− 0.555	0.026*			0.201	0.456	
SSAS		0.209	0.437			− 0.060	0.826	
TAS-20		0.214	0.427			− 0.108	0.692	

^b^ Spearman’s rank correlation

WL5, five-minute water load test; HTT, heartbeat tracking task; PDS, postprandial distress syndrome; PCS, physical component summary; MCS, mental component summary; HADS, Hospital Anxiety and Depression Scale; SSAS, Somatosensory Amplification Scale; TAS-20, Toronto Alexithymia Scale of 20 items

### Correlation between WL5, HTT, and other measures in the HV group

Table 4 shows the correlation coefficients for each of the measured items within the HV group. There was a moderate negative significant correlation between WL5 and HTT scores (r = − 0.509, P < 0.05) and a moderate negative significant correlation between WL5 and HADS-depression scores (r = − 0.524, P < 0.05). The HTT was not correlated with any other measurement, including HADS-anxiety, in the HV group.

**Table 4:** Spearman’s rank correlation between WL5, the HTT score, and other measures in HV

		WL5				HTT		
		r	P^b^			r	P^b^	
HTT		− 0.509	0.044*					
PCS		0.024	0.931			− 0.178	0.509	
MCS		0.106	0.696			0.434	0.093	
HADS-anxiety		− 0.460	0.073			− 0.061	0.823	
HADS-depression		− 0.524	0.037*			− 0.078	0.775	
SSAS		− 0.340	0.197			− 0.033	0.905	
TAS-20		− 0.103	0.704			− 0.362	0.168	

^b^ Spearman’s rank correlation

WL5, five-minute water load test; HTT, heartbeat tracking task; HV, healthy volunteers; PCS, physical component summary; MCS, mental component summary; HADS, Hospital Anxiety and Depression Scale; SSAS, Somatosensory Amplification Scale; TAS-20, Toronto Alexithymia Scale of 20 items

## Discussion

Our aim was to determine the relationship between WL5 drinking volume and HTT in PDS and HV groups. The results showed no significant difference in HTT performance between the groups; only the drinking water volume was significantly lower in the PDS group. In addition, the perceptual relationship between organs observed in HV was lost in patients with PDS.

In PDS, the cardiac-gastric perceptual relationship is lost and the interoceptive perceptual function of the stomach is abnormal. The relationship between the WL5 drinking volume and HTT score in HV was similar to that in previous studies [10, 11]. Patients with PDS showed hypersensitivity limited to the stomach, and the perceptual relationship between bodily axes that is present in HV was lost. Compared with patients with EPS, those with PDS have comparable brain activity during water loading, but resting activity is not a problem [23]. Irritable bowel syndrome (IBS), which is included in FGID, is also a syndrome of organ-specific hypersensitivity such as FD [19, 20], while IBS and healthy individuals show no difference in cardiac IAc [32]. Furthermore, it has been reported that heartbeat perception does not differ between healthy persons and those with fibromyalgia [33]. The findings of these previous studies were consistent with our results in that no hypersensitivity was found in the cardiac tissue, which is not a symptomatic site. Moreover, the results of the lost cardiac-gastric perceptual relationship observed in HV indicated that the interoceptive perceptual function of PDS patients may be altered. Although our study was unable to determine why this change in interoceptive perceptual function occurred, we consider several possibilities. Fibromyalgia is a disease that is thought to involve hypersensitivity; however, the hypersensitivity is limited to the threat modality and not to the innocuous organ of the heart [33]. It has also been reported that the satiety and bloating in FD patients are induced by cognitive influences [34]. As in fibromyalgia, changes in gastric interoceptive perception may occur in PDS due to a specific awareness regarding gastric sensations and eating.

The SSAS, a measure of severity of subjective visceral sensory hypersensitivity, did not differ between PDS and HV; previous studies have shown that FD tends to have mildly higher SSAS [35, 36], and our results differ from the SSAS characteristics assumed in FD patients. It is possible that this is due to the fact that PDS symptoms appear mainly as postprandial symptoms. Postprandial symptoms in FD are influenced by hypersensitivity to peripheral stretch rather than central influences such as subjective hypersensitivity [37]. The result that subjective sensitivity in PDS is not related to the volume of water loading is similar to that reported by Steinsvik. The loss of the cardiac-gastric perceptual relationship, the lack of a SSAS relationship, and the lack of any difference from HV indicates that the VH of PDS affects only the stomach, not all organs.

Testing WL5 and HTT simultaneously can be helpful in determining whether the VH is localized or widespread. Although testing across bodily axes has been examined in relation to gastric and rectal barostats, VH, and somatic pain thresholds, there has been no simple method of testing [17, 20]. We have identified different sensory perception relationships between bodily axes in patients with PDS and HV. The combination of two tests that can look at the relationship between perception of the stomach and different bodily axes provides clues to understanding whether VH with FD is localized or widespread.

Considering the negative correlation between drinking volume and depression in both PDS and HV, it is possible that gastric perception is influenced by specific emotional experiences in depressive mood states. The anterior cingulate cortex (ACC) is an important site in visceral sensory processing associated with emotional experiences [38]. The ACC is responsible for the processing of negative emotions and is involved in the activity patterns seen in depression [39, 40]. The ACC is activated by gastric balloon stimulation [38] and is involved in the emotionally motivated response to visceral pain in negative emotional states [41]. FD is associated with activation of the ACC, failure of AIC activation, and failure to integrate gastric sensation, and accompanying psychological states may affect gastric sensory processing [24, 42]. Our results showed a similar negative relationship between the stomach and heart in HV who exhibited significantly lower HADS-depression scores than patients with PDS. This suggests that depressive symptoms as well as negative emotional states may affect gastric sensations, especially satiety and thresholds for extensor stimulation.

### Future directions

Patients with EPS may show a different perceptual relationship between bodily axes than those with PDS; EPS has a stronger central hyperactivity at rest than PDS, which is involved in its pathogenesis [23]. It has been suggested that the difference between localized or widespread VH may be a difference in the intensity of central versus peripheral pathology [20]. In the future, it may be possible to clarify the difference in pathophysiology between EPS and PDS by examining the relationship between cardiac IAc and gastric perception in patients with EPS as well. Second, it may be necessary to examine the involvement of comorbid psychiatric symptoms in patients with FD as well as their multifaceted emotional state in hypersensitivity. Because the present study used the HADS, which focuses on symptoms, it was not possible to determine whether specific emotions are involved in stomach perception. Compared with anxiety and depression, the association between negative emotional states that are not symptoms and sensory perception has not been the topic of much research.

### Limitations

First, the method for measuring IAc in the gastric region is underdeveloped, and the water loading test may be influenced by gastric accommodation, adaptive relaxation, and gastric emptying. Second, we did not measure sensory perception in other bodily axes, such as the respiratory system. Finally, our study has a small sample size and a single-center design, in addition to a sample with low depression and anxiety scores and no comorbid psychiatric disorders, which are common characteristics of patients with FD. Therefore, we must be cautious about generalizing our results.

## Conclusion

Patients with PDS showed visceral hypersensitivity limited to the stomach, and the perceptual relationships between organs observed in HV were lost. PDS may cause abnormal interoceptive perceptual function of sensory stimuli in the stomach; the combination of the WL5 and HTT, tests that examine sensation in different bodily axes, is useful in assessing whether VH is localized or widespread.

## Data Availability

The datasets used and analyzed during the current study are available from the corresponding author upon reasonable request.

## Abbreviations

ACC: anterior cingulate cortex
AIC: anterior insular cortex
EPS: epigastric pain syndrome
FD: functional dyspepsia
FGID: functional gastrointestinal disorder
HADS: Hospital Anxiety and Depression Scale
HRQoL: Health-related quality of life
HTT: heartbeat tracking task
HV: healthy volunteers
IAc: interoceptive accuracy
IBS: irritable bowel syndrome
MCS: mental component summary
PCS: physical component summary
PDS: postprandial distress syndrome
SD: standard deviation
SSAS: Somatosensory Amplification Scale
TAS-20: Toronto Alexithymia Scale of 20 items
VH: visceral hypersensitivity
WL5: five-minute water load test
